# Supplementary material for: Effects of Statins on Lipid Profile of Kidney Transplant Recipients: A Meta-Analysis of Randomized Controlled Trials
Source: Biomed Res Int. 2020 May 2;2020:9094543. doi: 10.1155/2020/9094543 (PMC7212277; doi:10.1155/2020/9094543)
Supplement: Supplementary Materials — The following two results are, respectively, Embase and SinoMed search strategies. [file 9094543.f1.docx]

The following two results are respectively Embase and SinoMed search strategies.

## Embase Session Results

| No. | Query | Results |
| --- | --- | --- |
| #19 | #4 AND #18 | **4501** |
| #18 | #5 OR #6 OR #7 OR #8 OR #9 OR #10 OR #11 OR #12 OR #13 OR #14 OR #15 OR #16 OR #17 | **319905** |
| #17 | 'hydroxymethylglutaryl‐coa reductase inhibitors' | **527** |
| #16 | 'hmg-coa reductase inhibitors'/exp OR 'hmg-coa reductase inhibitors' | **147025** |
| #15 | 'lovastatin'/exp OR lovastatin | **15922** |
| #14 | 'hypercholesterolemia'/exp | **71221** |
| #13 | 'hyperlipidemia'/exp | **154579** |
| #12 | 'dyslipidemia'/exp | **65993** |
| #11 | 'hydroxymethylglutaryl coenzyme a reductase inhibitor'/exp | **146851** |
| #10 | 'pravastatin'/exp | **19308** |
| #9 | 'fluindostatin'/exp | **9123** |
| #8 | 'simvastatin'/exp | **36131** |
| #7 | 'atorvastatin'/exp | **35270** |
| #6 | 'cerivastatin'/exp | **3756** |
| #5 | 'rosuvastatin'/exp | **14098** |
| #4 | #1 OR #2 OR #3 | **157825** |
| #3 | 'acute renal allograft rejection' | **654** |
| #2 | 'renal transplantation' | **42398** |
| #1 | 'kidney transplantation'/exp | **152087** |

No. Query

Sinomed result

1) "lovastatin" [not weighted: extension] OR "hydroxymethyl glutaric acyl CoA reductase inhibitors" [not weighted: extension] OR "atorvastatin calcium" [not weighted: extension] OR "rosuvastatin calcium "[unweighted: extended] 9812

2) " kidney transplantation "[unweighted: extended] 17216

3) (#2) AND (#1) 12
